# Assessment of the Circulating PD-1 and PD-L1 Levels and P53 Expression as a Predictor of Relapse in Pediatric Patients with Wilms Tumor and Hypernephroma

**DOI:** 10.3390/children11091035

**Published:** 2024-08-23

**Authors:** Heba A. Sahyon, Nadaa S. Alharbi, Zummar Asad, Mohamed A. El Shishtawy, Safaa A. Derbala

**Affiliations:** 1 Chemistry Department, Faculty of Science, Kafrelsheikh University, Kafrelsheikh 33516, Egypt; 2 Department of Medicine & Surgery, Royal College of Surgeons in Ireland, D02 YN77 Dublin, Ireland; nadaaalharbi@rcsi.ie (N.S.A.); zummarasad@rcsi.ie (Z.A.); 3 Ministry of Health, Riyadh 12233, Saudi Arabia; 4 Forensic Medicine and Clinical Toxicology Department, Faculty of Medicine, Benha University, Benha 13518, Egypt; mohamed.alsheshtawy@fmed.bu.edu.eg; 5 Urology, and Nephrology Center, Mansoura University, Mansoura 35516, Egypt; safaaderbala@mans.edu.eg

**Keywords:** Wilms tumor, hypernephroma, pediatric renal tumor, PD-1, PD-L1, P53, relapse

## Abstract

**Background/Objectives:** Wilms tumor (WT) is the most common form of pediatric renal tumor, accounting for over 90% of cases followed by hypernephroma. Some pediatric patients with WT (10%) experience relapse or metastasis and have poor survival rates. PD-L1 assists cancer cells in escaping damage from the immune system. P53 mutations are found in relapsed WT tumor samples. We hypothesized that testing circulating PD-1 and PD-L1 and P53 expression levels could offer a simple method to predict patient relapse and explore novel treatments for pediatric WTs and hypernephroma. **Methods:** Flow cytometric detection of cPD-1, cPD-L1, and P53 expression in relapsed and in-remission WT and hypernephroma before and after one year of chemotherapy was performed. **Results:** Our data shows increased levels of cPD-L1 in relapsed pediatric patients with WT or hypernephroma before and after chemotherapy. There were also slight and significant increases in cPD-1 levels in relapsed groups before chemotherapy. Additionally, we observed significant decreases in P53 expression after one year of chemotherapy in relapsed pediatric patients. **Conclusions:** Our study found that circulating PD-L1 can be used as a predictor marker for WT and hypernephroma relapse. In conclusion, these circulating markers can assist in monitoring relapse in WT and hypernephroma patients without the need for several biopsies.

## 1. Introduction

Pediatric renal tumors can affect newborns, kids, teens, and young adults and are broadly categorized as benign or malignant. The clinical presentation of these tumors is influenced by age, tumor type, and disease stage. Hematuria, fever, anorexia and weight loss, constipation, abdominal/flank pain, and, in some cases, respiratory compromise are common symptoms [1]. Pediatric renal tumors are highly diverse and account for 3.2–11.1% of juvenile cancer cases worldwide. Wilms tumor (WT) is the most common form of pediatric renal tumor, accounting for over 90% of cases. Other, less common forms include congenital mesoblastic nephroma (CMN), renal medullary carcinoma (RMC), pediatric renal cell carcinoma (RCC) or hypernephroma, clear cell sarcoma (CCS) of the kidney, and malignant rhabdoid tumor (MRT). However, some pediatric patients with WT (10%) experience relapse or metastasis and have poor survival rates [2,3]. Several cytokine expressions in the tumor tissues of WT patients are directly correlated with metastasis. These cytokines, namely, IL-6, TGF-β1, and IFN-γ, are associated with poor prognosis and recurrence of WT [4,5]. Hypernephroma is a more prevalent kidney cancer than WT in children aged over 10 years. Hypernephroma is caused when cells in the lining of the kidney’s tubules proliferate uncontrollably. Hypernephroma is an aggressive cancer that quickly spreads to adjacent organs and the lungs [6].

Peripheral blood mononuclear cells (PBMCs) have emerged as a noninvasive marker for identifying important gene expressions that can predict and indicate certain malignant diseases such as pancreatic cancer and renal cell carcinoma [7,8,9]. The gene expressions in PBMCs show promising potential for early cancer detection because differences in their expression can be observed as soon as the cancer becomes immunogenic or starts evading the immune system [10,11,12].

The immune microenvironment significantly impacts the development of tumors. However, some solid tumors, such as lung cancer and melanoma, have seen significant treatment advancements with immunotherapy drugs [13]. These drugs are successful in treating solid tumors that have higher tumoral programmed death ligand 1 (PD-L1) expression [14]. PD-L1 is the main ligand of programmed death 1 (PD-1), which is a co-inhibitory receptor that can be constitutively expressed or induced in myeloid, lymphoid, normal epithelial cells, and cancer. Cancer cells can escape from damage by the immune system through the PD-L1 receptor interacting with the PD-1 on immune cells [15]. Multiple studies have found that various malignant tumors abnormally express PD-L1, indicating a possible connection to poor prognosis [16,17,18,19,20,21,22]. Few studies have been conducted to evaluate the immunohistochemical PD-L1 expression in WT [23,24,25,26]. Their results have shown that PD-L1 expression is linked to WT recurrence and progression, which makes it a potential predictive biomarker for patients’ prognosis.

P53 is a crucial gene related to cancer. It is known as a tumor suppressor gene, and mutations in this gene are linked to many human cancers. Proper P53 function controls the cell cycle, while mutations cause irregular mitotic division and increased DNA content [27]. P53 mutations are the most common change observed between primary and relapse tumor samples from patients with WT [28]. Multiple studies have shown that detecting P53 by immunostaining is strongly related to anaplasia in WT [29,30,31,32]. Moreover, overexpression of p53 is closely linked to anaplasia in WT and is considered a prognostic factor [33]. Also, the overexpression of p53 is linked to a poorer prognosis and more advanced clinicopathological characteristics in individuals with renal cell carcinoma, suggesting that p53 could be a potential target for RCC treatment [34]. Serum-soluble P53 levels were detected in hepatocellular carcinoma [35], cervical cancer [36], and breast cancer [37] patients. It was established that P53 was highly expressed in PBMCs in inflammation or malignancy [38,39]. Until now, no study has detected P53 protein expression in the peripheral blood mononuclear cells of WT pediatric patients to correlate it with the clinical outcomes of these patients.

Elevated oxidative stress may lead to genetic instability, triggering the development of malignancies. P53 plays a crucial role in managing how cells respond to oxidative stress. The activation of p53 can stimulate genes that control the production of antioxidants such as catalase and superoxide dismutase (SOD), helping to alleviate the impact of oxidative stress [40]. Increased oxidative stress can lead to the enhanced expression of immune checkpoint proteins, such as PD-1 and PD-L1, in the tumor area. The interaction between PD-1 and PD-L1 is vital in suppressing the body’s immune response to tumors, enabling cancer cells to evade detection and elimination by the immune system [41]. Therefore, cancer cells produce a high level of free radicals.

However, tumor biopsy testing is not favorable for WT pediatric patients. Therefore, detecting soluble proteins for the purpose of detecting WT’s prognosis or recurrence is necessary. It has been found that PD-1 and PD-L1 are detected either in tissue or soluble forms [42,43]. Also, it has been revealed that aggressive renal cell carcinoma patients have elevated levels of serum soluble PD-1 (sPD-1), which is associated with advanced stages [44]. sPD-L1 can be detected in the sera of clear cell renal cell carcinoma patients and might impair host immunity systemically, in turn promoting cancer progression and resulting in poor clinical outcomes [45]. The process of measuring PD-1 and PD-L1 expressions on the cell surface is intricate. However, determining the level of their soluble form could offer a comparatively uncomplicated approach to promptly gathering information regarding patients’ conditions. To the best of our knowledge, no study has investigated the circulating PD-1, PD-L1, (cPD-1, cPD-L1), and P53 expression levels in WT and hypernephroma pediatric patients or their correlation to relapse prediction.

The interaction between oxidative stress markers, the tumor suppressor gene, and immune checkpoint proteins (PD-1, PD-L1) is complex and diverse within the context of cancer. Understanding these connections can offer valuable knowledge about the processes involved in cancer development and advancement, as well as possible targets for cancer treatment. Therefore, this study aimed to investigate the correlation between cPD-1, cPD-L1 levels, P53 expression, oxidative stress markers (malondialdehyde, catalase, SOD), and the likelihood of relapse in children with Wilms tumors and hypernephroma. The goal was to help oncologists find new treatment approaches for pediatric WTs and hypernephroma to prevent future relapses.

## 2. Materials and Methods

### 2.1. Subjects

Pediatric cases who were admitted to Tanta Oncology Center were diagnosed and treated between October 2022 and January 2024. The current study consists of 50 pediatric patients with renal cancer (age 0.33–13 years), divided into 25 with Wilms tumors, 17 with renal cell carcinoma (hypernephroma), and 8 with clear cell sarcoma (CCS). All cases experienced primary nephrectomy (unilateral total nephrectomy) followed by chemotherapy according to the hospital’s protocol. This study involved two groups of children: in-remission cases and relapsed cases. The in-remission cases were selected from children who had been treated for WT or hypernephroma and had not experienced recurrence or progression during the follow-up period. Relapsed cases were selected from children who had received chemotherapy but experienced a recurrence or progression of the tumor within a year after treatment. Several cases were excluded from the study, including pediatric patients who underwent surgery without requiring chemotherapy, those with focal or diffusely anaplastic histology, those with metastatic cancer cells in the histological examination of the safe margin, and those with stage IV or V who had metastatic or bilateral WT. Additionally, the study did not include any cases that discontinued chemotherapy prematurely for any reason.

### 2.2. Sampling Method

The study included pediatric cases who were diagnosed at the Tanta Oncology Centre and had full clinicopathological reports. All selected patients had initial diagnoses of kidney tumors confirmed by pathology and underwent surgery without any prior chemotherapy or radiation therapy. All patients received chemotherapy for 27–34 weeks based on tumor stage and patient health. According to the hospital protocol, pediatric patients were followed up after one year of starting the treatment. Blood samples were taken one week before the chemotherapy and after one year of starting the chemotherapy. All blood samples were taken routinely in the hospital’s laboratory and used in this study.

The Ethics Committee of the Benha University, Faculty of Medicine, approved the study (REC) (No. RC.19.10.2022). Consent was obtained from the guardian of each child whose specimens were included, and the privacy of each child was strictly maintained. The research process adhered to the principles outlined in the Declaration of Helsinki.

### 2.3. Methods

All tested parameters were assessed in patients’ heparinized whole blood before and after one year of chemotherapy.

#### 2.3.1. Flow Cytometry Investigations

##### Sample Preparations

Blood samples were taken from the vein using vacutainers containing EDTA. The peripheral blood mononuclear cells (PBMCs) were then separated from the blood by density-gradient centrifugation for 20 min at 1500 rpm, using Ficoll-Paque Plus solution. The bands of cells were carefully transferred to another centrifuge tube filled with phosphate buffer saline (PBS) or Hank’s solution and mixed. The lymphocyte sediment was then fixed with ice-cold absolute alcohol (1 mL) and stored at 4 °C until analysis.

Before testing, a volume of 200 µL of lymphocytes was transferred to plain test tubes washed with 1 mL of PBS and then centrifuged at 200 rpm for 5 min. After centrifugation, the supernatant was discarded, and the lymphocyte pellets were mixed well with PBS and centrifuged again. The washed pellets were then mixed with the required antibody for *PD-1* or *PD-L1* (3 µL) and incubated at room temperature for 20 min. After incubation, the cells were washed with 1 mL of PBS and the tubes were centrifuged at 2000 rpm for 5 min. The supernatant was removed, and the cells were fixed with 200 µL of 4% paraformaldehyde for further analysis by flow cytometry (BD FACScanTM system was used for flow cytometry, Becton Dickinson, SanJose, CA, USA).

In the PBMCs, the detection of P53 involves experiencing DNA damage and other stresses, leading to a range of post-translational modifications that cause its stabilization, accumulation in the nucleus, and activation as a transcription factor. Although the majority of p53 gathers in the nucleus following stress, there remains a considerable percentage in the cytoplasm, which also acts in tumor suppression [46]. In this study, P53 staining was performed as reported in the method of Cavalcantithe et al., 2011 [47]. The washed PBMC was soaked in a solution of saponin for permeabilization (Thermo Scientific Chemicals, Waltham, MA, USA) for 10 min at room temperature. Following this period, the cells were rinsed twice with PBS containing 0.05% Tween 20 (Merck, Darmstadt, Germany) and utilized for staining with anti-FITC p53 using the direct staining method. Next, 100 µL of cell suspension and 10 µL of mouse anti-FITC p53 were incubated in the dark for 10 min, then washed twice with PBS/BSA to eliminate excess fluorescence and subsequently analyzed using flow cytometry. Fluorescent histograms were generated from gated events exhibiting side and forward light-scattering characteristics of viable cells.

#### 2.3.2. Flow Cytometric Parameters

The activity of *PD-1* or *CD279* was measured using a PE mouse anti-human CD279 (*PD-1*) antibody (Cat. No.560795) according to the manufacturer’s instructions. The activity of *PD-L1* or *CD274* was measured using the RY586 mouse anti-human CD274 (B7-H1) antibody (Cat. No.753201) according to the manufacturer’s instructions. The *P53* protein levels were evaluated by flow cytometry using mouse anti-FITC p53 (Cat. No. 554218, BD Biosciences, Bicton, Dickinson, CA, USA).

#### 2.3.3. Antioxidant Parameters

Malondialdehyde (MDA), catalase, and superoxide dismutase were assessed in the plasma of the heparinized whole blood of all cases using ELISA kits (RayBiotech, Peachtree Corners, GA, USA). These tests were carried out following the manufacturer’s guidelines. The activity of SOD in each sample was calculated in U/mL, while MDA was expressed as nmol/m1, and catalase was expressed as mM.

#### 2.3.4. Statistical Analysis

SPSS-PC software version 20 was employed for the statistical calculations. Parametric data were expressed as mean ± standard deviation (S.D.), and comparisons between groups were made using a one-way ANOVA test. Comparisons between non-parametric groups were made using the Mann–Whitney U test and data were expressed as median and range.

## 3. Results

The demographic characteristics of all pediatric patients are listed in Table 1. The current study included fifty pediatric cases of kidney cancer: 23 males (46%) and 27 females (54%) with ages from 0.33 to 13 years. In pediatric cases, 15 patients were relapsed after 1 year (8 cases with WT and 7 cases with hypernephromas), whereas no patients were relapsed in CCS. Table 1 shows no significant differences between males and females in age or sex in the WT and CCS groups. However, most diagnoses in the hypernephroma pediatric group were female—11 (64.70%). Furthermore, 21 (84%) of the WT pediatric patients were under six years old, and 4 (16%) were over six. Comparably, all 17 hypernephroma patients (100%) were over six years old. This distribution was also seen in CCS patients, with 7 (87.5%) under six years old and only 1 (12.5%) over six years old (Table 1). Among these pediatric patients, 14 were diagnosed with stage III (32%, 29.41%, and 12.5%) of WT, hypernephroma, and CCS, respectively. The percentages of pediatric patients presenting with stage II were 32%, 17.65%, and 12.5% for WT, hypernephroma, and CCS, respectively. Furthermore, the percentages for stage I were 36%, 52.9%, and 75% for WT, hypernephroma, and CCS, respectively (Table 1). In terms of tumor origin, WT cases originated from the left kidney in 17 (68%) of children, whereas for hypernephroma, the majority originated from the right kidney in 10 (58.8%) of cases. Meanwhile, CCS patients showed that the majority originated from the left kidney in six cases (75%). When it comes to anaplasia, 22 (88%) of WT cases, 15 (88.2%) of hypernephroma cases, and 8 (100%) of CCS cases exhibited anaplasia. Finally, the majority of the pediatric patients with WT, hypernephroma, and CCS came from rural areas, with percentages of 84%, 70.6%, and 62.5%, respectively (Table 1).

### 3.1. Elevated cPD1 and cPD-L1 Levels Were Correlated to Relapsed Patients

**Figure 1:**
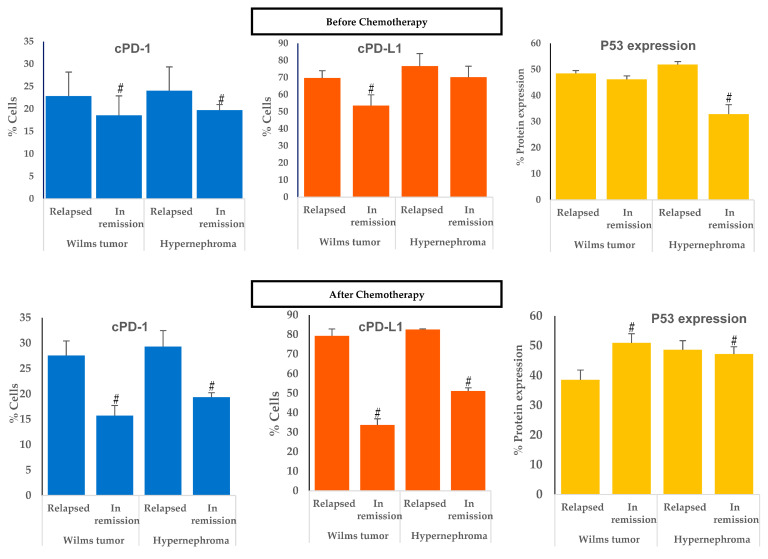
Circulating PD-1, PD-L1, and P53 expression levels before and after one year of chemotherapy between in-remission and relapse groups. ^#^ *p* ≤ 0.05 represents a significant difference compared to relapsed group.

**Figure 2:**
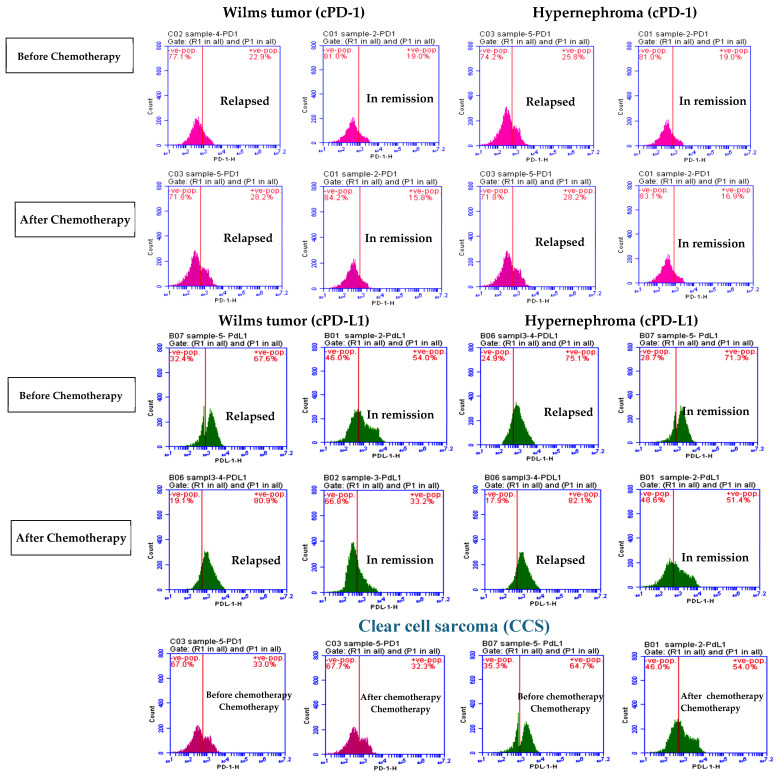
Circulating PD-1 and PD-L1 levels in relapsed and in-remission study groups before and after one year of chemotherapy.

Based on a literature survey that revealed detectable levels of sPD-1 and sPD-L1 in specimens of cancer patients with different types of cancer [48,49,50,51,52], in our study, we evaluated cPD-1 and cPD-L1 in pediatric patient specimens diagnosed with WT, hypernephroma, and CCS by flow cytometry. Moreover, we compared the levels of cPD-1 and cPD-L1 in the relapsed patients’ group with those in remission in the pediatric patients’ group. Figure 1 demonstrates significant reductions in cPD-1 levels in the in-remission group compared to the relapsed group before chemotherapy in pediatric patients with Wilms’ tumors (*p* = 0.046) and hypernephroma (*p* = 0.023) (Figure 2). However, there was no significant difference in cPD-L1 levels detected between the relapsed hypernephroma pediatric patients’ group before chemotherapy and those in remission (*p* = 0.083, Figure 2). Furthermore, after chemotherapy, there were still significant decreases in both cPD-1 and cPD-L1 levels in in-remission pediatric patients with WT and hypernephroma compared to those who experienced relapse (*p* ≤ 0.0001 for all).

According to Table 2, significant increases in both cPD-1 and cPD-L1 levels were observed in the relapsed group of pediatric patients with WT after one year of chemotherapy compared to before chemotherapy (*p* = 0.043 and 0.001, respectively) (Figure 2). On the contrary, there were significant decreases in both the cPD-1 and cPD-L1 levels of the in-remission WT pediatric patients after one year of chemotherapy compared to before chemotherapy (*p* = 0.022, and 0.001, respectively).

**Figure 3:**
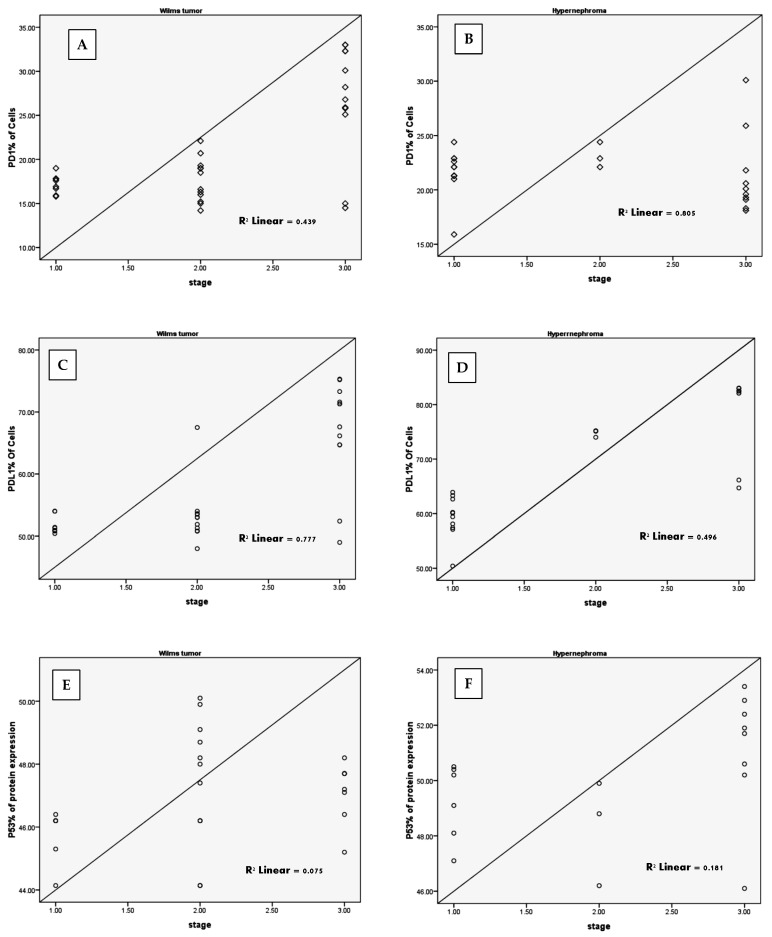
Circulating PD-1, PD-L1, and P53 expression correlations to kidney tumor stage in WT (**A**,**C**,**E**) and hypernephroma (**B**,**D**,**F**) groups.

There was a positive correlation between cPD-1 and cPD-L1 and the tumor stage in WT pediatric patients (Figure 3A,C). No correlation was observed in pediatric patients with hypernephroma for cPD-1 (Figure 3B), but there was one observed for cPD-L1 and tumor stage (Figure 3D). Furthermore, there were no correlations between cPD-1, cPD-L1, and the tumor stage in WT or hypernephroma pediatric patients (Figure 3E,F, respectively). Furthermore, significant increases in both cPD-1 and cPD-L1 levels were observed in the relapsed group of hypernephroma pediatric patients after one year of chemotherapy compared to before chemotherapy (*p* = 0.044 and 0.039, respectively), while the in-remission group showed no significant difference in cPD-1 levels before and after chemotherapy (Table 2). On the contrary, there was a significant decrease in the cPD-L1 of the in-remission hypernephroma pediatric patients after one year of chemotherapy compared to before chemotherapy (*p* = 0.001). Lastly, it was noticed that there was no significant difference in cPD-1 levels among CCS in-remission pediatric patients after one year of chemotherapy compared to their levels before chemotherapy (*p* = 0.182). However, there was a significant decrease observed in cPD-L1 levels after one year of chemotherapy compared to the levels before chemotherapy (*p* = 0.001, Table 2).

Based on our data, patients who experienced relapse had higher levels of both cPD-1 and cPD-L1 before receiving chemotherapy, with cPD-L1 levels showing a more significant increase. This indicates that detecting elevated cPD-L1 levels before treatment could potentially serve as a dependable indicator of relapse. This information might be utilized by oncologists to adjust the treatment plan. Furthermore, elevated levels of cPD-L1 after chemotherapy might suggest a higher probability of cancer recurrence.

### 3.2. Elevated P53 Concentration in Relapsed Patients

**Figure 4:**
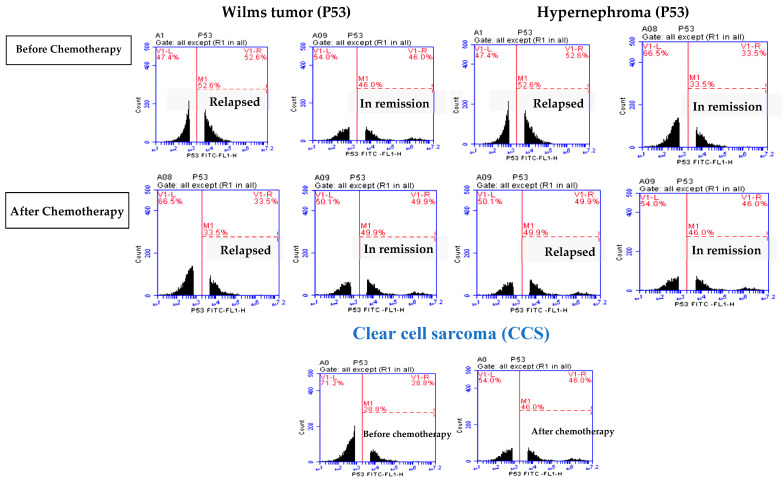
P53 expression levels in relapsed and in-remission study groups before and after one year of chemotherapy.

In Figure 1, no significant difference is shown in P53 expression levels between the WT relapsed and in-remission groups before chemotherapy. However, there was a significant increase in P53 expression levels in the relapsed hypernephroma pediatric group before chemotherapy compared to the remission group (*p* = 0.001). Additionally, after one year of chemotherapy, there was a significant decrease in the P53 expression level in relapsed WT pediatric patients compared to their levels in the in-remission group (*p* ≤ 0.0001). In contrast, after one year of chemotherapy, the P53 expression level in the pediatric group with hypernephroma in remission was significantly decreased compared to its level in the relapsed group (*p* = 0.001, Figure 4). Additionally, after one year of chemotherapy in CCS pediatric patients, the expression level of P53 was found to be significantly higher compared to its levels before the treatment (*p* = 0.001, Table 2, and Figure 4). As listed in Table 2, there were significant decreases in P53 expression levels in pediatric patients with WT and hypernephroma who relapsed after one year of chemotherapy compared to their levels before chemotherapy (*p* = 0.001 and 0.002, respectively). On the other hand, there were significant increases in P53 expression levels in in-remission pediatric patients with WT and hypernephroma after one year of chemotherapy compared to their levels before chemotherapy (*p* = 0.001 and 0.001, respectively). These findings suggest that the P53 expression level may not be a reliable predictor of relapse before starting the treatment, but it could be used to evaluate cancer recurrence if it decreases after the completion of chemotherapy.

### 3.3. Oxidative Stress Markers in Relapsed Pediatric Patients

The elevated superoxide dismutase activity, along with the reduced catalase activity observed in Wilms’ tumors, could lead to an overproduction of hydrogen peroxide. As a result, both enzymes may sensitize rather than protect this tumor from oxidative stress [53]. In this study, all patients had undergone surgical removal of cancers, which increased their lipid peroxidation markers (MDA) before chemotherapy. However, there were no significant differences found in MDA levels between the WT and hypernephroma relapsed and in-remission groups before chemotherapy (Table 3). Furthermore, after one year of chemotherapy, there were significant decreases in the MDA levels of the WT and hypernephroma in the in-remission pediatric groups compared to their levels in the relapsed ones (*p* ≤ 0.0001 for all). Conversely, after one year of chemotherapy, there were significant increases in both catalase and SOD activities in the in-remission WT pediatric group compared to the relapsed one (*p* = 0.003 and 0.009, respectively). Similarly, after one year of chemotherapy, there were significant increases in both catalase and SOD activities in the in-remission WT pediatric group compared to the relapsed one (*p* = 0.0001 and 0.036, respectively). On the other hand, after one year of chemotherapy, there were significant decreases in the in-remission WT, hypernephroma, and CCS pediatric patients compared to their levels before chemotherapy (*p* = 0.001, 0.001, and 0.002, respectively). This decrease in MDA levels may be attributed to the removal of cancer burden in the in-remission pediatric patients, which balanced the ROS production and decreased the lipid peroxidation marker. In contrast, the MDA levels in the relapsed WT and hypernephroma patients did not change after one year of chemotherapy compared to its levels before chemotherapy (Table 3). This may be due to cancer recurrence, which increases the oxidative stress again. Furthermore, after one year of chemotherapy, the catalase and SOD levels in relapsed WT and hypernephroma pediatric patients were significantly decreased compared to their levels before treatment. This decrease may have been due to increased ROS production from the recurrent tumor, which consumed these enzymes, leading to their depletion. In contrast, the catalase and SOD levels in the in-remission WT, hypernephroma, and CCS pediatric patients’ groups were significantly increased after one year of chemotherapy compared to their levels before treatment (Table 3). These data confirmed the removal of cancer burden in the in-remission groups, which increased both SOD and catalase levels to rebalance the ROS production that may have been increased by chemotherapy.

## 4. Discussion

In our study, many pediatric patients under six years in the WT and CCS groups were affected (84% and 87.5%, respectively). However, in hypernephroma, all affected children were over six years old (100%). This age group is the most affected by WT in children, as indicated in previous studies [54,55,56]. The gender distribution in our study is almost equal, while other studies have shown a higher ratio of female diagnoses with kidney tumors. Conversely, other studies have shown a higher rate of affected males compared to females [54,57]. We attribute our gender ratio to the small sample size in our study and the selected criteria that excluded late-stage patients. In our study, approximately half of the pediatric patients (48%) were diagnosed with stage I disease, followed by stage III (28%). This contrasts with other studies that have reported stages III-V in pediatric patients, which may be due to our exclusion criteria for stages beyond III. The high percentage of WT, hypernephroma, and CCS pediatric patients without anaplasia (88%, 88.2%, and 100%, respectively) correlates with findings from similar studies [54,58]. Relatively, only 12% of WT and 11.8% of hypernephroma pediatric patients were diagnosed with anaplasia, percentages lower than those of a study that reported about 6.1% anaplastic WT pediatric patients [59], but quite similar to another study reporting 10% anaplastic WT [60]. This percentage may be due to delays in diagnosis, as most pediatric patients were from rural areas. The kidney tumors in our patient groups were unilateral in all cases, mostly originating from the right side in hypernephroma (58%) and mainly from the left side in WT pediatric patients (68%). The origin of these kidney tumors is consistent with a Nigerian study where WT mainly originated in the left kidney [58], but is consistent with another study as well [54].

As far as we know, this is the first study to determine the predicting significance of circulating PD-1 and PD-L1 levels and the P53 protein expression in pediatric kidney tumor patients. PD-1 and PD-L1 are two proteins that contribute to the growth of cancer cells by interfering with the immune response. PD-1, also called CD279, is expressed on activated immune cells and is highly expressed on tumor-specific T cells [61]. On the other hand, PD-L1, also called CD279 and B7-H1, is usually expressed by macrophages and some activated immune cells [62]. Tumor cells were found to have increased levels of PD-L1, which helped them evade the immune system’s surveillance and promote cancer growth [22]. When PD-L1 binds to its receptors, it activates signaling pathways that promote tumor growth. This discovery suggests that PD-L1 plays a role in the progression of tumors [63]. Furthermore, PD-L1 has been found to have non-immune proliferative effects on various types of tumor cells. For instance, PD-L1 has been observed to trigger epithelial-to-mesenchymal transition and stem cell-like characteristics in renal cancer cells, which suggests that the intrinsic pathway of PD-L1 facilitates the progression of kidney cancer [64]. Additionally, numerous studies have examined PD-L1 biomarker expression in cancer biopsies as a means of predicting relapse and survival rates [24,25,26]. These studies have found that PD-L1 expression is linked with late-stage WT and unfavorable histology, which means that PD-L1 expression predicts a poor prognosis. Nevertheless, none of these studies have established that PD-L1 expression is a predictive factor for relapse and/or decreased survival. Moreover, PD-1 and PD-L1 were found to be soluble in serum as well as attached to cell membranes [42,43]. However, it has been found that in patients with gastric cancer, the levels of serum PD-L1 and the expression of tissue PD-L1 can potentially be used as biomarkers to predict the chances of recurrence and to determine the prognosis [65]. Our data show that relapsed WT or hypernephroma pediatric patients have increased levels of cPD-L1 before chemotherapy. Interestingly, these levels continue to increase after one year of chemotherapy, indicating that cancer cells express PD-L1 in their cells to evade the immune system. Recent studies have confirmed that there is an increase in PD-L1 expression in WT kidney tissues [24,66], as well as increased plasma levels of PD-L1 [67]. Also, it was noticed that increased PD-L1 expression in the kidney tissues of children with WT at different tumor stages could be a predictor for chemotherapy failure [25]. Consistent with these data, it was observed in our data that both relapsed WT and hypernephroma pediatric patients had slight and significant increases in cPD-1 levels before chemotherapy. These levels continued to increase even after a year of chemotherapy. These data were consistent with Frigola et al. [44], who noticed that elevated levels of soluble PD-L1 in aggressive renal cell carcinoma patients could be a prognostic factor for cancer progression or even death. This may be due to PD-L1 in the cancer cells binding to the PD-1 receptor on tumor-specific T cells, which requires an increase in PD-1 levels to compensate for the rise in PD-L1 levels, allowing the cancer cells to evade the immune system. However, a study by Pinto et al., 2016 [68], revealed the immunohistochemical determination of both PD-1 and PD-L1 gives low expression in kidney tumor tissues, and this may be due to scoring methods. In contrast, our study indicated that the cPD-1 and cPD-L1 levels were opposed in in-remission WT, hypernephroma, and CCS pediatric patients as their levels were reduced after one year of chemotherapy, especially for the cPD-L1 levels. This could establish that cPD-1 levels are not a reliable predictor marker for relapse; however, cPD-L1 levels could be considered a reliable predictor marker for relapse.

Mutations in the p53 gene are almost exclusively found in advanced cancer types [69,70]. The detection of p53 through immunohistochemistry in WT can be used as a substitute marker for P53 mutation, which makes it easier to determine anaplasia [33,71,72]. P53 protein could be nuclear or cytoplasmic [73,74]. When DNA damage occurs, p53 expression is induced, and the protein is transferred to the nucleus to regulate gene expression directly [75]. Cytoplasmic repossession of P53 is thought to be an important mechanism for disrupting its function as a tumor suppressor. In 37% of breast cancers, P53 was found localized in the cytoplasm, indicating a mechanism of inhibiting P53 function through nuclear exclusion [76].

In cancer cells, the disturbance of p53 signaling pathways can lead to uncontrolled cell proliferation, avoidance of programmed cell death, and heightened genetic instability, all contributing to the development and advancement of tumors [27]. In PBMCs, p53 plays a crucial role in the cellular reaction to various forms of cellular stress and harm, such as DNA damage, oxidative stress, and inflammation. Upon identification of cellular damage, p53 can become active, resulting in the increased expression of genes involved in halting the cell cycle, repairing DNA, and inducing apoptosis [38]. The activation of p53 in PBMCs can induce an adaptive immune response because p53 can regulate the activity and maturation of immune cells such as lymphocytes and monocytes. The absence of p53 function in cancer is associated with the advancement of tumors, while in PBMCs, the activation of p53 is a part of the cellular defense response to various forms of cellular stress and harm [75]. A recent study confirmed the correlation between WT mixed histology and low expression of p53 [44]. However, older studies confirmed overexpression of p53 in WT tissues [31,33]. When exposed to different factors that cause cell death, P53 immediately relocates to the mitochondria. At the mitochondria, p53 initiates the permeabilization of the outer membrane, which results in the discharge of pro-apoptotic proteins. It has been observed that p53, when directed to the mitochondria, can impede the growth of tumors [77]. Our study has shown significant decreases in the expression of P53 in both WT and hypernephroma patients who relapsed after one year of chemotherapy. This reduction could be attributed to the cancer recurrence that inhibited the production of P53 [78]. However, the decreased expression levels of P53 in WT and hypernephroma pediatric patients before the treatment may have occurred because of the production of mutated P53, which acts to increase cancer proliferation instead of inhibition [79,80,81].

On the other hand, our study revealed that there were significant increases in the expression levels of P53 in WT, CCS, and hypernephroma pediatric patients who did not relapse after one year of chemotherapy. This increase in P53 could indicate the activation of P53, which works to inhibit cancer recurrence [82]. Thus, these higher expression levels of P53 may indicate successful cancer treatment. Our study’s findings suggest that, while the P53 expression level may not be a reliable predictor of relapse before starting treatment, it could be useful in evaluating cancer recurrence if it decreases after completing chemotherapy.

Cancer burden typically leads to an increase in reactive oxygen species, which arise from a high metabolic rate and contribute to damaging DNA and genomic instability, thus promoting cancer metastasis [83]. Moreover, the surgical removal of tumors in patients with Wilms tumors had led to an increase in oxidative stress due to elevated lipid peroxidation [84]. The differences in the functionality of direct antioxidant enzymes are typically evident as reduced catalase and superoxide dismutase activities in tumor tissues compared to the corresponding adjacent tumor-free tissue. In our study, the decreased SOD and catalase activities in the relapsed pediatric patient groups may have been due to the increased oxidative stress produced from cancer recurrence, which consumed these enzymes [85]. Also, increased SOD and catalase activities in the in-remission pediatric groups suggest a better cellular defense mechanism against oxidative stress, which could contribute to improved treatment outcomes. This indicates that enhancing antioxidant defenses might be an important factor in achieving and maintaining remission in pediatric WT and hypernephroma patients.

Our study found that circulating PD-L1 can be used as a predictor marker for cancer relapse. However, the limited number of patients studied prevents us from establishing a definitive conclusion. To overcome this limitation, we recommend studying a larger group of pediatric patients and regularly analyzing these circulating markers every month or two. This could help to identify early recurrence of kidney cancer and improve risk stratification, ultimately leading to better-targeted treatment. Also, this could ultimately improve survival rates without increasing the risk of relapse. Moreover, tumor cells may use the release of cPD-L1 molecules as a mechanism to hinder antitumoral responses throughout the body. Thus, clinically beneficial treatments may involve the deactivation or removal of circulating PD-L1 molecules by targeted drugs.

## 5. Conclusions

This study investigated the correlation between cPD-1, cPD-L1 levels, P53 expression, oxidative stress markers, and the likelihood of relapse in children with Wilms’ tumors (WTs) and hypernephroma. Our findings revealed that before chemotherapy, high levels of antioxidant enzymes SOD and catalase, along with overexpression of P53, led to increased production of cPD-L1 and cPD-1. This allowed cancer cells to evade the immune system. Elevated levels of cPD-L1 and cPD-1 were associated with weakened immune responses and cancer recurrence. Recurrent cancer cells exhibited increased cPD-L1 levels, likely due to the depletion of antioxidant enzymes and P53 suppression. Based on this, we can infer that monitoring cPD-L1 levels can help to identify patients at a higher risk of relapse and guide treatment strategies. This suggests that cPD-1 and cPD-L1 markers can assist in monitoring relapse in WT and hypernephroma patients without the need for multiple biopsies.

Our future research will include examining these markers in a large group of pediatric patients over short follow-up periods, such as one or two months, as well as for a longer time. That study will aim to identify the survival rate and compare these markers to the histological data obtained from biopsies to validate the use of these markers as an alternative to biopsies.

## Figures and Tables

**Table 1:** Demographic data of the studied groups.

	WT (n = 25)	Hypernephroma (n = 17)	CCS (n = 8)
Age (year)			
Mean	3.78	5.7	3.22
Range	0.67–9.2	0.33–13	0.75–8
Sex			
Male	13 (52%)	6 (35.3%)	4 (50%)
Female	12 (48%)	11 (64.7%)	4 (50%)
Site			
Right	8 (32%)	10 (58.8%)	2 (25%)
Left	17 (68%)	7 (41.18%)	6 (75%)
Stages			
I	9 (36%)	9 (52%)	6 (75%)
II	8 (32%)	3 (17.6%)	1 (12.5%)
III	8 (32%)	5 (29.41%)	1 (12.5%)
Anaplasia			
With	3 (12%)	2 (11.76%)	
Without	22 (88%)	15 (88.24%)	8 (100%)
Location			
Rural	21 (84%)	12 (70.6%)	5 (62.5%)
Urban	4 (16%)	5 (29.4%)	3 (37.5%)

WT = Wilms tumor, and CCS = clear cell sarcoma, n represents sample size.

**Table 2:** Circulating PD-1, PD-L1, and P53 expression levels in relapsed/in-remission pediatric patients diagnosed with WT, hypernephroma, and CCS before and after one year of chemotherapy.

Parameters		Wilms Tumor		Hypernephroma		CCS In Remission (n = 8)
		Relapsed (n = 8)	In Remission (n = 17)	Relapsed (n = 7)	In Remission (n = 10)	
**cPD-1**	Before	22.85 ± 5.37	18.59 ± 4.33	24.07 ± 5.28	19.72 ±1.24	32.3 ± 1.3
	After	27.58 ± 2.85 *	15.73 ± 2.02 *	29.32 ± 3.17 *	19.38 ± 0.9	31.14 ± 0.77
**cPD-L1**	Before	69.76 ± 4.21	53.58 ± 6.27	76.68 ± 7.32	70.17 ± 6.45	69. 6 ± 6.25
	After	79.3 ± 3.57 *	33.74 ± 3.18 *	82.53 ± 0.37 *	51.12 ± 1.68 *	59.82 ± 1.8 *
**P53**	Before	48.5 ± 1.08	46.21 ± 1.33	51.87 ± 1.16	32.88 ± 3.6	28.86 ± 2.73
	After	38.55 ± 3.3 *	50.9 ± 3.10 *	48.64 ± 3.04 *	47.21± 2.43 *	46.65 ± 1.0 *

* *p* ≤ 0.05 represents a significant difference compared to before chemotherapy.

**Table 3:** MDA level, catalase, and SOD activities in relapsed/in-remission pediatric patients diagnosed with WT, hypernephroma, and CCS before and after one year of chemotherapy.

Parameters		Wilms Tumor		Hypernephroma		CCS (n = 8)
		Relapsed (n = 8)	In Remission (n = 17)	Relapsed (n = 7)	In Remission (n = 10)	
**MDA** nmol/mL	Before	198.9 ± 20	185.11 ± 38.78	401.28 ± 10.5	397.10 ± 39.1	159 ± 7.9
	After	198.37 ± 17.5	144.35 ± 20.50 *^#^	397.7 ± 13.1	174.5 ± 33.5 *^#^	141.25 ± 10.29 *
**Catalase** mM	Before	6.23 ± 0.15	5.52 ± 2.7	2.48 ± 0.27	2.84 ± 1.0	11.2 ± 1.3
	After	5.9 ± 0.5 *	7.43 ± 1.92 *^#^	2.09 ± 0.25	11.67 ± 1.6 *^#^	13.38 ± 1.2 *
**SOD** U/mL	Before	160.26 ± 6.1	159.67 ± 14.9	46.32 ± 16.3	39.72 ± 2.6	70.9 ± 6.7
	After	152.95 ± 11.3 *	166.68 ± 11.2 *^#^	36.73 ± 2.58 *	43.7 ± 8.69 *^#^	53.07 ± 9.8 *

* *p* ≤ 0.05 represents a significant difference compared to before chemotherapy. ^#^ *p* ≤ 0.05 represents a significant difference compared to relapsed group.

## Data Availability

The data presented in this study are available on request from the corresponding author due to ethical reasons.
